# Association between Posttreatment Serum Platelet-to-Lymphocyte Ratio and Distant Metastases in Patients with Hepatocellular Carcinoma Receiving Curative Radiation Therapy

**DOI:** 10.3390/cancers15071978

**Published:** 2023-03-26

**Authors:** Dong Soo Lee, Chang Wook Kim, Hee Yeon Kim, Young-Mi Ku, Yoo Dong Won, Su-Lim Lee, Der Sheng Sun

**Affiliations:** 1 Department of Radiation Oncology, College of Medicine, The Catholic University of Korea, Seoul 06591, Republic of Korea; 2 Division of Gastroenterology and Hepatology, Department of Internal Medicine, College of Medicine, The Catholic University of Korea, Seoul 06591, Republic of Korea; cwkim@catholic.ac.kr (C.W.K.); hee82@catholic.ac.kr (H.Y.K.); 3 Department of Radiology, College of Medicine, The Catholic University of Korea, Seoul 06591, Republic of Korea; ymiku@catholic.ac.kr (Y.-M.K.); yoodong1@catholic.ac.kr (Y.D.W.); radlsl@catholic.ac.kr (S.-L.L.); 4 Division of Oncology, Department of Internal Medicine, College of Medicine, The Catholic University of Korea, Seoul 06591, Republic of Korea; medi9652@catholic.ac.kr

**Keywords:** biomarker, hepatocellular carcinoma, metastasis, platelet-to-lymphocyte ratio, prognosis, radiation therapy, serum

## Abstract

**Simple Summary:**

In unresectable hepatocellular carcinoma (HCC), the use of definitive radiation therapy (RT) as a combined locoregional therapeutic strategy has been increasing. Distant metastasis (DM) is one of the main causes of the declining quality of life and survival rates in the majority of cancer patients, necessitating the clinical research of key DM indicators. On the other hand, various serum biomarkers and indices of immune and inflammatory status have been investigated. This study aimed to investigate whether serum immune and inflammatory parameters can help to predict DM in HCC. In our comprehensive evaluation, the highest value of the posttreatment platelet-to-lymphocyte ratio and the lowest value of the posttreatment prognostic nutritional index were significant independent prognostic indicators of distant control and overall survival rates, respectively. Future research is necessary to confirm our findings.

**Abstract:**

Background: We sought to investigate whether serum immune and inflammatory parameters can help to predict distant metastasis (DM) in patients with unresectable hepatocellular carcinoma (HCC) undergoing curative radiation therapy (RT). Methods: A total of 76 RT courses were analyzed. The following variables were included in the analysis: systemic inflammation index, neutrophil-to-lymphocyte ratio, platelet-to-lymphocyte ratio (PLR), prognostic nutritional index (PNI), absolute lymphocyte count, lymphocyte-to-monocyte ratio, albumin, albumin-to-alkaline phosphatase ratio, RT-related parameters, and levels of total protein, hemoglobin, α-fetoprotein, and PIVKA-II. Distant control (DC) and overall survival (OS) rates were calculated and compared. Results: The mean age was 61.4 years, and most patients were men (*n* = 62, 81.6%). The median RT fraction number and fractional doses were 12 (range, 4–30) and 5 (range, 2–12) Gy, respectively. With a median follow-up of 12 (range, 3.1–56.7) months, the 1-year DC and OS rates were 64.4% and 55.2%, respectively. The development of DM significantly deteriorated OS (*p* = 0.013). In the multivariate analysis, significant independent prognostic indicators for DC and OS rates were the highest posttreatment PLR (≤235.7 vs. >235.7, *p* = 0.006) and the lowest posttreatment PNI (≤25.4 vs. >25.4, *p* < 0.001), respectively. Conclusions: Posttreatment serum PLR might be helpfully used as a predictive biomarker of DM in unresectable HCC patients undergoing RT. Future research is necessary to confirm our findings.

## 1. Introduction

The incidence and mortality rates for hepatocellular carcinoma (HCC) are increasing worldwide [1,2,3]. According to cancer statistics reported in 2021, the overall incidence and mortality rates decreased from 1999 to 2018 in Korea [4]. Among both men and women, the 5-year relative survival rates increased from 11.8% in 1993–1995 to 37% in 2014–2018 [4].

In unresectable HCC, the use of radiation therapy (RT) as a combined locoregional therapeutic strategy after arterially directed therapies has been increasing [5,6,7,8,9]. Recent advances in RT technologies have improved the delivery of higher doses to the tumor while sparing normal tissues in the surrounding area [10,11,12,13,14,15].

Various serum biomarkers and indices of immune and inflammatory status have been investigated for solid malignancies [16,17,18,19,20,21,22]. The prognostication of cancer outcomes based on serum indices has the clinical strengths of being simple, cost-effective, and repeatable [23]. Representative serum indices associated with cancer prognosis include the lymphocyte-to-monocyte ratio (LMR), neutrophil-to-lymphocyte ratio (NLR), platelet-to-lymphocyte ratio (PLR), prognostic nutritional index (PNI), and systemic inflammation index (SII). However, the results are conflicting between studies, and no firm conclusions have been made [19,24,25].

On the other hand, distant metastasis (DM) is one of the main causes of declining quality of life and survival rates in the majority of cancer patients [26,27,28]. Therefore, identifying key indicators of DM can be a crucial component of clinical research.

Recent evidence suggests that a high PLR may be related to early distant recurrence, poor overall survival, and tumor aggressiveness [20,25,29,30,31,32,33,34,35,36,37]. Here, we investigated whether serum markers are useful for the prediction of clinical outcomes (especially for DM) in patients with unresectable HCC receiving curative RT in the contemporary era.

## 2. Materials and Methods

### 2.1. Study Population

Between January 2014 and April 2019, RT was administered to the liver in a total of 95 courses in 89 HCC patients. The inclusion criteria for the study were a diagnosis of unresectable HCC by radiological or serological diagnostic criteria and receipt of RT with a potentially curative intent at doses of ≥30 Gy. The exclusion criteria were (a) receipt of RT with palliative intent at doses < 30 Gy, (b) lack of completion of the planned RT course, (c) loss to follow-up within 3 months after treatment, (d) lack of data for serum laboratory testing before, during, or after treatment, and (e) the presence of metastatic disease. After applying the inclusion and exclusion criteria, a total of 76 RT courses in 71 patients were included and analyzed in this study.

### 2.2. Definition of Serum Indices and Data Collection

The LMR, NLR, PLR, PNI, SII, absolute lymphocyte count (ALC), concentrations of albumin (A), albumin-to-alkaline phosphatase ratio (AAR), total protein (P) and hemoglobin (H) were calculated according to the following equations.

LMR = lymphocyte count [10^9^/L]/monocyte count [10^9^/L]

NLR = neutrophil count [10^9^/L]/lymphocyte count [10^9^/L]

PLR = platelet count [10^9^/L]/lymphocyte count [10^9^/L]

PNI = (5 × lymphocyte count [10^9^/L]) + (10 × albumin [g/dL])

SII = neutrophil count [10^9^/L] × platelet count [10^9^/L]/lymphocyte count [10^9^/L]

ALC = absolute lymphocyte count [10^9^/L]

A = 10 × albumin [g/dL]

AAR = 10 × albumin [g/dL]/alkaline phosphatase [IU/L]

P = 10 × total protein [g/dL]

H = hemoglobin [g/dL].

All serum indices obtained before, during, and after treatment were assessed to improve the accuracy of the study. For pretreatment indices, serum samples taken just before treatment (RT) were analyzed. For posttreatment indices, all serum indices during and after treatment (RT) were collected. We did not assure whether the lowest or highest value for each index would be significantly associated with the study end points. Therefore, for the serial serum data, we recorded both the lowest and highest values of serum indices. We collected posttreatment data up to 3 months after the completion of treatment in consideration of the systemic posttreatment effects of RT. In addition, pretreatment levels of α-fetoprotein (AFP) and PIVKA-II were collected.

### 2.3. Treatments

The combination treatment approach was applied in most of the study population. This involved curative-aimed RT followed by several cycles of transarterial chemoembolization (TACE) or hepatic arterial infusion chemotherapy (HAIC) because of incomplete TACE or HAIC.

Various methods for RT were used. Because of movement according to the respiratory cycle, RT simulation was performed with four-dimensional computed tomography (CT). RT targets and organs-at-risk were contoured according to the definition of the International Commission on Radiation Units and Measurements (ICRU) reports 50, 62, and 83 [38,39]. Thereafter, the RT technique was selected based on the patients’ liver function and performance status, tumor extent and size, and tumor motion. Comparative plan evaluation was conducted to choose the most appropriate treatment method if clinically needed using the Eclipse treatment planning system (ECLIPSE™, Varian Medical Systems, Palo Alto, CA, USA). RT was delivered using 6–10 megavoltage photons. After RT, subsequent therapies comprising TACE/HAIC were applied if additional treatments were required based on imaging studies.

### 2.4. Study End Points

After completion of RT, imaging studies were conducted using contrast-enhanced CT scanning at 1 month and Primovist-enhanced magnetic resonance imaging at 3 months to assess the tumor response. Patients were usually followed every 1–3 months after RT and more frequently if clinically needed. Follow-up data were collected and recorded in the patients’ electronic medical charts.

Distant control (DC) and overall survival (OS) duration were defined as follows: DC as the interval from the last date of RT to the date of distant metastasis outside the liver or the date of last follow-up; and OS as the interval from the last date of RT to the date of death by any causes or the date of last follow-up.

As secondary study end points, we also calculated local control (LC) and intrahepatic control (IHC) rates and investigated the effect of these factors on OS duration.

To determine the follow-up time in patients who received two courses of RT, the date of the last follow-up in the first course was defined as the first date of the second course of RT.

### 2.5. Statistical Analysis

The data were analyzed using R statistical software (version 4.0.2; R Foundation for Statistical Computing, Vienna, Austria) and SPSS Statistics (version 12.0; SPSS Inc., Chicago, IL, USA). Descriptive statistics and patient demographics were analyzed to identify characteristics that predicted outcomes. The Kolmogorov–Smirnov test was used to test for normality. Comparison of values between two groups was conducted using the Wilcoxon rank sum test. To identify the optimal cutoff values for the study end points, the maximal chi-square test was applied using R statistics and verified using SPSS statistics. The Kaplan–Meier method was used for survival analysis, and survival graphs were compared using the log-rank test. To assess the prognostic significance, the multivariate Cox proportional hazards model was used. A *p*-value < 0.05 was regarded as significant.

## 3. Results

### 3.1. Patient and Tumor Characteristics

A total of 76 RT courses among 71 patients were analyzed for patients treated between January 2014 and April 2019. The baseline patient and tumor characteristics are described in Table 1. The mean age was 61.4 years and most were men (*n* = 62, 81.6%). Most of the study population had an Eastern Cooperative Oncology Group performance status scale of 0 or 1 (*n* = 73, 96.1%). Forty (52.6%) patients had combined comorbidities such as cerebrovascular accident, chronic kidney disease, chronic obstructive pulmonary disease, coronary artery disease, diabetes mellitus, or hypertension. The most common etiology of HCC was hepatitis B virus (*n =* 43, 56.6%) followed by mixed type (*n* = 12, 15.8%), and alcoholic (*n* = 10, 13.2%). Portal vein or inferior vena cava thrombosis was combined in 32 (42.1%) patients. The Child–Turcot–Pugh score was categorized as Class A (5 or 6) in 55 (72.4%), Class B (7–9) in 20 (26.3%), and Class C (10) in 1 (1.3%) patient.

### 3.2. Treatment Characteristics

Most of the study population underwent pre-RT TACE/HAIC (*n* = 74, 97.4%). In patients who received pre-RT TACE/HAIC, the median number of treatment cycles was three (range, 1–20). RT was performed using either three-dimensional conformal RT (3-D CRT) (*n* = 14, 18.4%), gating stereotactic body RT or intensity-modulated RT (IMRT) (*n* = 26, 34.2%), volumetric modulated arc therapy (*n* = 25, 32.9%), or multifield static IMRT (*n* = 11, 14.5%). The median RT fraction number and fractional doses were 12 (range, 4–30) and 5 (range, 2–12) Gy, respectively. The median biologically equivalent dose by α/β = 10 was 72.6 (range, 51.5–119) Gy. Using the Eclipse treatment planning system, we calculated gross tumor volume (GTV), total liver volume (TLV), total liver dose (TLD), and liver dose (LD) (total liver volume minus the planning target volume (PTV)). The median GTV and TLV, mean TLD, and mean LD (TL-PTV) were 55.3 (range, 2.8–1288) cc, 1260.6 (range, 551–2559.8) cc, 2,150.2 (range, 327.5–4142.9) cGy, and 1615 (range, 253.4–2993.9) cGy, respectively. Treatment characteristics are summarized in Table 2.

### 3.3. Distribution of Serum Indices

The numerical distributions of the pre- and posttreatment serum indices are shown in Table 3. For pretreatment indices, one value just before RT was collected. For the indices during and after treatment, as previously described, we included both the highest and lowest values (except for ALC) among all serial serum data recorded. The distribution of serum indices according to DM is depicted in Supplementary Table S1.

### 3.4. Survival Analysis and Effect on Overall Survival

With a median follow-up of 12 (range, 3.1–56.7) months, the 1-year and 2-year OS rates were 55.2% and 33.1%, respectively. The 1-year and 2-year DC, LC, and IHC rates were 64.4% and 53.6%, 88.2% and 85.7%, and 50.5% and 28.1%, respectively. During the follow-up period, death, DM, local recurrence (LR), and intrahepatic recurrence (IHR) occurred in 52 (68.4%), 29 (38.2%), 8 (10.5%), and 40 (52.6%) patients, respectively.

**Figure 1:**
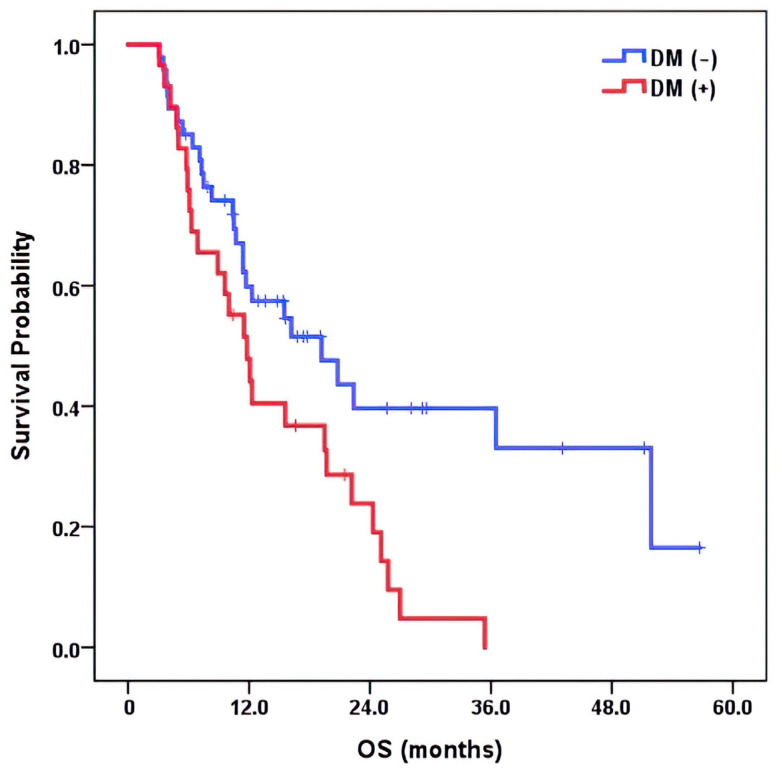
Kaplan–Meier OS curve according to the occurrence of DM.

In the Cox proportional hazards model, development of DM significantly deteriorated OS rates (*p* = 0.013), while LR (*p* = 0.691) and IHR (*p* = 0.106) did not (Figure 1).

### 3.5. Determination of the Optimal Cutoff Points for Variables

Using the maximal chi-square statistical method, we calculated the cutoff points and *p*-values for major study end points to identify the most suitable cutoff values that would maximally separate the Kaplan–Meier curves (Table 4). Post-PLR-H (H: highest) (*p* = 0.014) and GTV (*p* = 0.035) were significant prognosticators for DC rates. For OS, Post-SII-H (*p* = 0.047), Post-PLR-H (*p* = 0.017), Post-PNI-L (L: lowest) (*p* = 0.001), Post-A-L (*p* = 0.002), Post-P-L (*p* = 0.006), and Post-H-L (*p* = 0.024) were significant prognosticators.

### 3.6. Multivariate Prognostic Factor Analysis

**Figure 2:**
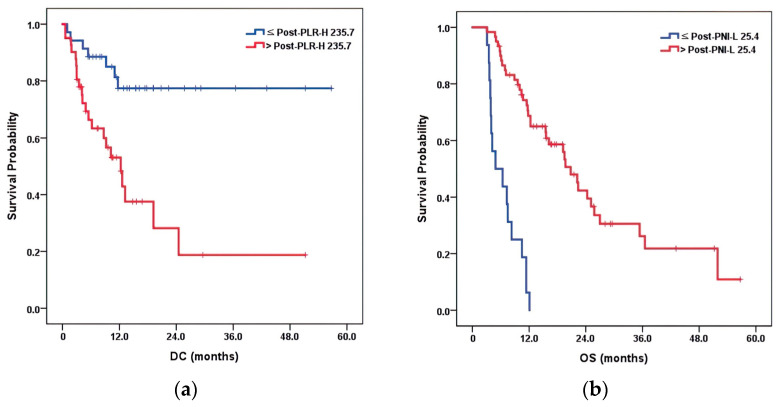
Kaplan–Meier curves for DC (**a**) and OS (**b**).

The results of the multivariate Cox proportional hazards analysis for the study end points are shown in Table 5. Post-PLR-H (*p* = 0.006) for DC and Post-PNI-L (*p* < 0.001) for OS remained significant prognostic factors in the multivariate analysis. The 1-year probability of each group was as follows: for DC, 77.5% for Post-PLR-H ≤ 235.7 vs. 53.1% for Post-PLR-H > 235.7; and for OS, 0.63% for Post-PNI-L ≤ 25.4 vs. 68.7% for Post-PNI-L > 25.4. The total numbers of events in each group were seven in PLR-H ≤ 235.7 vs. 22 in Post-PLR-H > 235.7 in terms of DM, and 16 in Post-PNI-L ≤ 25.4 vs. 36 in Post-PNI-L > 25.4 in terms of deaths. Kaplan–Meier curves for these variables are shown in Figure 2.

## 4. Discussion

In unresectable or locally advanced HCC, the prognosis is usually dismal despite the use of combined therapeutic approaches [5,40,41]. Moreover, numerous causes of complications and deaths can be connected with survival outcomes. Therefore, at the time of this investigation, we did not ensure that we could identify meaningful serological prognostic indicators that predicted survival and recurrence. However, we tried to examine all conceivable pre- and posttreatment serum parameters comprehensively and found that some serum indices significantly predicted the study end points.

A number of immune-related or inflammatory indices and markers measured in serum have been addressed and have demonstrated prognostic significance [16,17,18,19,20,21,22]. The liver is responsible for a variety of functions in the body, including glycogen storage, plasma protein synthesis, and drug detoxification [42]. The liver is also associated with the production of monocytes, which comprise 4–8% of all white blood cells, and plays a major role in immunological effects through the action of the reticuloendothelial system [43]. Therefore, the serial process of carcinogenesis in HCC and the responses to radiation by the liver might cause unique hemodynamic changes during the RT course, and meaningful parameters that predict significant end points will be usefully applied if these parameters can be validated.

The inflammatory response is increasingly recognized as aiding tumor formation, progression, and metastasis in a variety of ways [44]. Growing evidence suggests that systemic immune and inflammatory cells, such as lymphocytes, monocytes, neutrophils, and platelets, play important roles in cancer development through multiple pathways, including tumor initiation, proliferation, invasion, and migration [45,46]. Lymphocytes, especially tumor-infiltrating lymphocytes, play a pivotal role in antitumor immune effects by triggering cytotoxic cell death and inhibiting tumor proliferation and migration by secreting cytokines such as IFN-γ and TNF-α [23,47]. Thus, lymphopenia is recognized as a biological indicator of reduced immune surveillance [48]. Monocytes are recruited to tumor sites, where they differentiate into tumor-associated macrophages (TAMs) and divide into M2 macrophages, which have a poor antigen-presenting capacity and contribute to the suppression of Th1 adaptive immunity via locally produced chemokines or cytokines [49]. TAMs secrete forceful proangiogenic factors such as VEGF and TNF-α, which facilitate tumor-associated angiogenesis and aid tumor cell proliferation, migration, and metastasis [23,49]. In addition, TAMs suppress the antitumor response by producing IL-10 and prostaglandin E2 and promote tumor cell invasion and metastasis by producing MMP-2 and MMP-9 [49]. Neutrophils may facilitate tumor cell invasion by directly degrading the extracellular matrix and assisting the evasion of cancer cells from immune surveillance by impeding the cytolytic activity of lymphocytes and other immune cells [23,50]. Platelets are increasingly recognized as playing critical roles in cancer pathogenesis. Platelets protect circulating tumor cells (CTCs) from antitumor immune responses by producing TGF-β and adenine nucleotides, which stimulate the epithelial–mesenchymal transition and tumor cell extravasation, and thereby promote CTC metastasis [51,52]. In addition, platelets activated by cancer cells can induce phenotypic changes in cancer cells that foster their metastasis and angiogenesis [53]. Therefore, based on these concepts, the potential prognostic value of several immune or inflammatory parameters is obtaining a rational basis.

In the present study, the highest Post-PLR (Post-PLR-H) was the significant parameter associated with DM and an independent prognostic factor of DC rates. The association between a high PLR and poor prognosis has been demonstrated in several types of solid tumors [20,22,25,29,30,31,32,33,35,36,54]. However, treatment types, including immunotherapy, chemotherapy, RT, or surgery, varied among studies, and the majority of the studies focused on the pretreatment value of PLR. Very recently, a relationship between high PLR and distant organ metastasis in various types of cancers including breast, esophageal, hepatocellular, prostate, and thyroid cancer has been reported [32,33,34,35,55]. Our study results also support this theory of a robust role of PLR in the development of DM. In several studies, high PLR was related to not only aggressive tumor characteristics such as high-risk stage, lymph node metastasis, or vascular invasion but also poor DM-free survival, relapse-free survival, and OS [20,25,29,31,32,34,36,55]. In a review and meta-analysis by Li et al. [20], elevated pretreatment PLR was associated with reduced OS (HR = 1.45, 95% CI, 1.31–1.61) and progression-free survival (HR = 1.73, 95% CI, 1.31–2.29) in patients with advanced cancer. Bae et al. [30] showed that pretreatment platelet count, PLR, and posttreatment worsening of NLR, PLR and LMR were associated with IHR among patients receiving curative RT. PNI, particularly the lowest Post-PNI (Post-PNI-L), was significantly associated with OS in multivariate analysis. PNI has shown prognostic value regardless of the tumor origin [56,57,58,59,60,61,62], especially in gastrointestinal malignancies [58,63,64,65,66]. Nutritional impairment is associated with diminished performance, shorter survival, and increased mortality in cancer patients [67,68,69], and this is particularly concerning in patients with liver cancer given the presence of associated underlying diseases and the possibility of nutritional impairment due to cirrhosis [64]. Additionally, the composition of PNI (albumin and lymphocytes) may have a unique influence in HCC patients with limited survival duration. We need further research to elucidate the underlying mechanism concerning why particular (the highest or the lowest) values in posttreatment PLR and PNI were significantly connected to the study end points.

In terms of the effect on OS, the occurrence of DM significantly worsened OS rates, while LR and IHR did not. This result supports that DM is the major determinant of OS rather than recurrence within the liver. Therefore, finding serum markers associated with DM can be crucial from a clinical perspective.

The main strengths of the present study are the comprehensive assessment of serum-based parameters during all peri-treatment periods including the lowest and highest values, not merely confined to before or after treatment as performed in other prior studies. The role of PLR (Post-PLR-H) as an indicator of DM in HCC seems to be a new finding. Moreover, the PNI (Post-PNI-L) showed consistent predictive performance even in the population with limited survival outcomes. Our study also has some limitations. There was clinical heterogeneity in the study population, and the follow-up duration was relatively short owing to the limited survival duration of the patients. In addition, we did not evaluate treatment-related toxicity because that was not one of the study aims. Our analysis of numerus serum samples at different serial time points, calculations of each parameter, selection of the lowest and highest values for all parameters, and estimation of the optimal cutoff points were labor intensive. Recently, progressed computational algorithms have been able to help simplify the works and can validate the results in future studies [70].

## 5. Conclusions

In summary, serum-based indices independently predicted DC and OS rates in patients with unresectable HCC receiving curative RT. In addition, the occurrence of DM was the major determinant of OS. The highest posttreatment PLR (≤235.7 vs. >235.7) and the lowest posttreatment PNI (≤25.4 vs. >25.4) were significant prognostic factors for DC and OS rates, respectively. Appropriate selection of the most suitable cohorts and applicability of these parameters to HCC require further validation.

## Figures and Tables

**Table 1:** Baseline patient and tumor characteristics.

Characteristics		*n* (%)
Age		
Mean ± SD	61.4 ± 10.6	
Gender		
	Male	62 (81.6)
	Female	14 (18.4)
ECOG PS		
	0	30 (39.5)
	1	43 (56.6)
	2	3 (3.9)
Comorbidity		
	No	36 (47.4)
	Yes	40 (52.6)
HCC etiology		
	Alcoholic	10 (13.2)
	HBV	43 (56.6)
	HCV	4 (5.3)
	NBNC	4 (5.3)
	Mixed	12 (15.8)
	Unknown	3 (3.9)
Combined PVT/IVT		
	No	44 (57.9)
	Yes	32 (42.1)
C-T-P score		
	5 (A)	37 (48.7)
	6 (A)	18 (23.7)
	7 (B)	12 (15.8)
	8 (B)	5 (6.6)
	9 (B)	3 (3.9)
	10 (C)	1 (1.3)
AFP (ng/mL)		
Mean ± SD	709.5 ± 1808.9	
PIVKA-II (mAU/mL)		
Mean ± SD	3996.2 ± 18160	

ECOG PS, Eastern Cooperative Oncology Group performance status; HCC, hepatocellular carcinoma; HBV, hepatitis B virus; HCV, hepatitis C virus; NBNC, non-B and non-C; PVT, portal vein thrombosis; IVT, inferior vena cava thrombosis; C-T-P, Child–Turcot–Pugh; AFP, alpha-fetoprotein.

**Table 2:** Treatment characteristics.

Characteristics		*n* (%)
Pre-RT TACE/HAIC		
	No	2 (2.6)
	Yes	74 (97.4)
RT method		
	3-dimensional CRT	14 (18.4)
	Gating SBRT/IMRT	26 (34.2)
	Arc	25 (32.9)
	sIMRT	11 (14.5)
RT fraction number		
Median (range)	12 (4–30)	
RT fractional dose (Gy)		
Median (range)	5 (2–12)	
	≤5 Gy	53 (69.7)
	>5 Gy	23 (30.3)
Total BED_10_ (Gy)		
Median (range)	72.6 (51.5–119)	
GTV sum (cc)		
Median (range)	55.3 (2.8–1288)	
Total liver volume (cc)		
Median (range)	1260.6 (551–2559.8)	
Mean total liver dose (cGy)		
Median (range)	2150.2 (327.5–4142.9)	
Mean liver dose (TL-PTV) (cGy)		
Median (range)	1615 (253.4–2993.9)	

RT, radiation therapy; CRT, conformal radiation therapy; SBRT, stereotactic body radiation therapy; IMRT, intensity-modulated radiation therapy; sIMRT, static intensity-modulated radiation therapy; TACE, transcatheter arterial chemoembolization; HAIC, hepatic arterial infusion chemotherapy; BED_10_, biologically equivalent dose by α/β = 10; GTV, gross tumor volume; TL, total liver; PTV, planning target volume.

**Table 3:** Numerical distribution of pre- and posttreatment serum indices.

Indices	Pre-SII	Pre-NLR	Pre-PLR	Pre-PNI	Pre-ALC	Pre-LMR	Pre-A	Pre-AAR	Pre-P	Pre-H
Mean ± SD	374 ± 347.8	2.6 ± 1.6	119 ± 69	41 ± 8.1	1.3 ± 0.7	2.8 ± 1.1	35.3 ± 5.2	0.1 ± 0.05	71.3 ± 9.3	12.7 ± 1.8
**Indices**	**Post-SII-H**	**Post-SII-L**	**Post-NLR-H**	**Post-NLR-L**	**Post-PLR-H**	**Post-PLR-L**	**Post-PNI-H**	**Post-PNI-L**	**Post-ALC-L**	
Mean ± SD	1251.4 ± 1501.3	201.8 ± 194.7	14.8 ± 30.5	3.4 ± 10.1	320 ± 340	90.7 ± 36.6	40.5 ± 5.6	31.4 ± 6.3	0.4 ± 0.2	
**Indices**	**Post-LMR-H**	**Post-LMR-L**	**Post-A-H**	**Post-A-L**	**Post-AAR-H**	**Post-AAR-L**	**Post-P-H**	**Post-P-L**	**Post-H-H**	**Post-H-L**
Mean ± SD	3.1 ± 2.2	0.8 ± 0.4	36.7 ± 4.9	28.2 ± 5.8	1 ± 7.8	0.7 ± 5.3	73.3 ± 9.1	59.7 ± 9.7	13.4 ± 1.8	10.7 ± 2.1

SII, systemic inflammation index; NLR, neutrophil-to-lymphocyte ratio; PLR, platelet-to-lymphocyte ratio; PNI, prognostic nutritional index; ALC, absolute lymphocyte count; LMR, lymphocyte-to-monocyte ratio; A, albumin; AAR, albumin-to-alkaline phosphatase ratio; P, total protein; H, hemoglobin; SII-H, highest systemic inflammation index; SII-L, lowest systemic inflammation index; NLR-H, highest neutrophil-to-lymphocyte ratio; NLR-L, lowest neutrophil-to-lymphocyte ratio; PLR-H, highest platelet-to-lymphocyte ratio; PLR-L, lowest platelet-to-lymphocyte ratio; PNI-H, highest prognostic nutritional index; PNI-L, lowest prognostic nutritional index; ALC-L, lowest absolute lymphocyte count; LMR-H, highest lymphocyte-to-monocyte ratio; LMR-L, lowest lymphocyte-to-monocyte ratio; A-H, highest albumin; A-L, lowest albumin; AAR-H, highest albumin-to-alkaline phosphatase ratio; AAR-L, lowest albumin-to-alkaline phosphatase ratio; P-H, highest total protein; P-L, lowest total protein; H-H, highest hemoglobin; H-L, lowest hemoglobin.

**Table 4:** Optimal cutoff points of variables for major study end points.

Indices		Pre-SII	Pre-NLR	Pre-PLR	Pre-PNI	Pre-ALC	Pre-LMR	Pre-A	Pre-AAR	Pre-P	Pre-H
DC											
	*p*-value	0.756	0.910	0.874	0.596	0.390	0.690	0.511	0.841	1	0.885
	Cut-point	391.4	1.6	68.9	37.7	1.8	1.8	34	0.06	71	12.4
OS											
	*p*-value	0.968	0.552	0.896	0.075	0.514	0.187	0.283	0.285	0.737	0.522
	Cut-point	391.4	3.2	118.9	36.9	0.7	1.8	36	0.1	74	13.6
**Indices**		**Post-SII-H**	**Post-SII-L**	**Post-NLR-H**	**Post-NLR-L**	**Post-PLR-H**	**Post-PLR-L**	**Post-PNI-H**	**Post-PNI-L**	**Post-ALC-L**	
DC											
	*p*-value	0.277	0.080	0.105	0.082	**0.014**	0.358	0.489	0.180	0.176	
	Cut-point	804.8	288.3	4	1.9	235.7	94.7	37.2	38.6	0.6	
OS											
	*p*-value	**0.047**	0.570	0.213	0.825	**0.017**	0.898	0.060	**0.001**	0.495	
	Cut-point	426.9	277.9	3.8	3.1	235.7	68.2	41.2	25.4	0.6	
**Indices**		**Post-LMR-H**	**Post-LMR-L**	**Post-A-H**	**Post-A-L**	**Post-AAR-H**	**Post-AAR-L**	**Post-P-H**	**Post-P-L**	**Post-H-H**	**Post H-L**
DC											
	*p*-value	0.114	0.0857	0.479	0.699	0.618	0.900	0.806	0.975	0.134	0.846
	Cut-point	4	0.9	34	25	0.1	0.04	78	59	13.4	10.8
OS											
	*p*-value	0.196	0.065	0.069	**0.002**	0.419	0.102	0.916	**0.006**	0.844	**0.024**
	Cut-point	4.2	0.7	39	32	0.08	0.07	68	66	14.8	12.3
**Indices**		**AFP**	**PIVKA-II**	**Pre-CPTS**	**GTV sum**	**Total BED_10_**	MLD*	MLD**			
DC											
	*p*-value	0.101	0.165	0.790	**0.035**	0.808	0.272	0.700			
	Cut-point	47.4	24	5	504.7	67.5	2071.9	950.2			
OS											
	*p*-value	0.214	0.288	0.473	0.712	0.975	0.975	0.963			
	Cut-point	3.5	15	6	77.2	63.5	3458.8	1953.1			

DC, distant control; OS, overall survival; SII, systemic inflammation index; NLR, neutrophil-to-lymphocyte ratio; PLR, platelet-to-lymphocyte ratio; PNI, prognostic nutritional index; ALC, absolute lymphocyte count; LMR, lymphocyte-to-monocyte ratio; A, albumin; AAR, albumin-to-alkaline phosphatase ratio; P, total protein; H, hemoglobin; SII-H, highest systemic inflammation index; SII-L, lowest systemic inflammation index; NLR-H, highest neutrophil-to-lymphocyte ratio; NLR-L, lowest neutrophil-to-lymphocyte ratio; PLR-H, highest platelet-to-lymphocyte ratio; PLR-L, lowest platelet-to-lymphocyte ratio; PNI-H, highest prognostic nutritional index; PNI-L, lowest prognostic nutritional index; ALC-L, lowest absolute lymphocyte count; LMR-H, highest lymphocyte-to-monocyte ratio; LMR-L, lowest lymphocyte-to-monocyte ratio; A-H, highest albumin; A-L, lowest albumin; AAR-H, highest albumin-to-alkaline phosphatase ratio; AAR-L, lowest albumin-to-alkaline phosphatase ratio; P-H, highest total protein; P-L, lowest total protein; H-H, highest hemoglobin; H-L, lowest hemoglobin; AFP, alpha-fetoprotein; CPTS, Child–Turcot–Pugh score; GTV, gross tumor volume; BED_10_, biologically equivalent dose by α/β = 10; MLD*, mean liver dose; MLD**, mean liver (total liver volume minus the planning target volume) dose. Bold numbers indicate statistically significant values.

**Table 5:** Results of multivariate Cox proportional hazards analyses for major study end points.

End-Points	Variables	*p*-Value	Group	HR (95% CI)	1-Year Probability (%)
DC					
	Post-PLR-H	0.006	≤235.7	0.286 (0.117–0.700)	77.5
			>235.7	1	53.1
	GTV sum	0.068	≤504.7	0.413 (0.160–1.067)	68.1
			>504.7	1	28.6
OS					
	Post-SII-H	0.875	≤426.9	1	73
			>426.9	1.076 (0.435–2.662)	47.2
	Post-PLR-H	0.096	≤235.7	0.552 (0.275–1.110)	72.9
			>235.7	1	40.2
	Post-PNI-L	<0.001	≤25.4	4.790 (2.253–10.184)	0.63
			>25.4	1	68.7
	Post-A-L	0.136	≤32	1.963 (0.809–4.759)	45.1
			>32	1	80.7
	Post-H-L	0.735	≤12.3	1.188 (0.438–3.226)	45
			>12.3	1	92.9
	Post-P-L	0.256	≤66	1.981 (0.609–6.444)	49.1
			>66	1	79.4

DC, distant control; OS, overall survival; PLR-H, highest platelet-to-lymphocyte ratio; GTV, gross tumor volume; SII-H, highest systemic inflammation index; PNI-L, lowest prognostic nutritional index; A-L, lowest albumin; H-L, lowest hemoglobin; P-L, lowest total protein.

## Data Availability

The data are available upon request from the corresponding author.

## Supplementary Materials

The following supporting information can be downloaded at: https://www.mdpi.com/article/10.3390/cancers15071978/s1, Table S1: Distribution of serum indices according to distant metastasis.
