# Supplementary material for: Association between Posttreatment Serum Platelet-to-Lymphocyte Ratio and Distant Metastases in Patients with Hepatocellular Carcinoma Receiving Curative Radiation Therapy
Source: Cancers (Basel). 2023 Mar 26;15(7):1978. doi: 10.3390/cancers15071978 (PMC10092989; doi:10.3390/cancers15071978)
Supplement: Supplementary file 1 [file cancers-15-01978-s001.zip › cancers-2271748-supplementary.pdf]

Supplementary Table S1. Distribution of serum indices according to distant metastasis.

Serum indices	Pre			Post: High				Post: Low			
	DM (-) (n=47)	DM (+) (n=29)	*P*		DM (-) (n=47)	DM (+) (n=29)	*p*		DM (-) (n=47)	DM (+) (n=29)	*p*
SII			0.724	SII-H			0.295	SII-L			0.454
Mean±SD	374.5±381.3	373.1±291.7		Mean±SD	1162.7±1563.2	1395.2±1410.0		Mean±SD	184.9±169.8	229.1±230.1	
median(IQR)	249.9(151.3-458.5)	306.9(129.2-527.6)		median(IQR)	598.4(406.7-1281.4)	1024.7(406.8-1925.2)		median(IQR)	156.8(104.8-207.8)	187.5(92.3-301.5)	
NL			0.889	NL-H			0.146	NL-L			0.067
Mean±SD	2.8±1.9	2.4±1.0		Mean±SD	17.0±38.6	11.3±5.6		Mean±SD	4.0±12.8	2.4±0.9	
median(IQR)	2.2(1.4-3.4)	2.2(1.6-2.7)		median(IQR)	7.4(5.0-12.7)	9.8(6.4-14.7)		median(IQR)	1.7(1.5-2.6)	2.4(1.8-2.8)	
PL			0.94	PL-H			0.012	PL-L			0.104
Mean±SD	121.7±79.0	114.6±49.7		Mean±SD	298.5±391.6	354.8±235.9		Mean±SD	86.1±32.7	98.1±41.8	
median(IQR)	101.3(69.0-151.3)	104.4(78.6-148.7)		median(IQR)	217.3(157.4-291.1)	285.0(235.8-398.7)		median(IQR)	81.2(65.5-100.8)	95.7(78.8-109.7)	
PNI			0.05	PNI-H			0.074	PNI-L			0.248
Mean±SD	39.5±8.7	43.4±6.4		Mean±SD	39.6±5.8	41.9±4.9		Mean±SD	30.9±7.0	32.2±4.8	
median(IQR)	40.2(34.1-46.5)	42.5(39.7-48.4)		median(IQR)	39.5(34.4-44.7)	41.9(39.2-46.0)		median(IQR)	29.1(25.4-35.8)	34.1(29.0-36.0)	
ALC			0.494					ALC-L			0.081
Mean±SD	1.2±0.6	1.4±0.7						Mean±SD	0.5±0.3	0.4±0.2	
median(IQR)	1.0(0.8-1.6)	1.2(0.7-2.0)						median(IQR)	0.4(0.3-0.6)	0.3(0.2-0.4)	
LM			0.494	LM-H			0.748	LM-L			0.072
Mean±SD	2.7±1.1	2.9±1.1		Mean±SD	2.8±1.0	3.5±3.3		Mean±SD	0.9±0.5	0.7±0.3	
median(IQR)	2.7(2.0-3.4)	3.1(1.9-3.6)		median(IQR)	2.7(2.2-3.2)	2.5(2.2-3.5)		median(IQR)	0.9(0.5-1.1)	0.6(0.4-0.9)	
A			0.048	A-H			0.055	A-L			0.072

Mean±SD	34.3±5.5	36.8±4.4		Mean±SD	35.8±5.0	38.1±4.3		Mean±SD	27.3±6.4	29.6±4.5	
median(IQR)	34.0(30.0-39.0)	37.0(35.0-40.0)		median(IQR)	34.0(32.0-41.0)	39.0(35.0-41.0)		median(IQR)	26.0(22.0-33.0)	31.0(27.0-33.0)	
AA			0.773	AA-H			0.423	AA-L			0.724
Mean±SD	0.1±0.0	0.1±0.1		Mean±SD	1.6±9.9	0.1±0.1		Mean±SD	1.1±6.7	0.1±0.0	
median(IQR)	0.1(0.1-0.1)	0.1(0.1-0.2)		median(IQR)	0.1(0.1-0.2)	0.1(0.1-0.2)		median(IQR)	0.1(0.0-0.1)	0.1(0.0-0.1)	
P			0.626	P-H			0.548	P-L			0.898
Mean±SD	71.8±5.5	70.6±13.5		Mean±SD	73.6±4.7	72.8±13.5		Mean±SD	59.9±8.1	59.4±12.1	
median(IQR)	71.0(69.0-76.0)	73.0(69.0-75.0)		median(IQR)	74.0(72.0-77.0)	74.0(70.0-78.0)		median(IQR)	62.0(53.0-66.0)	61.0(57.0-65.0)	
H			0.352	H-H			0.037	H-L			0.273
Mean±SD	12.6±1.8	12.8±1.8		Mean±SD	13.1±1.8	13.9±1.7		Mean±SD	10.6±2.1	10.9±2.0	
median(IQR)	12.4(11.4-13.9)	12.9(11.5-14.1)		median(IQR)	13.1(11.5-14.5)	14.1(12.7-14.8)		median(IQR)	10.0(9.3-12.0)	11.3(9.9-12.3)	

DM, distant metastasis; SII, systemic inflammation index; NLR, neutrophil-to-lymphocyte ratio; PLR, platelet-to-lymphocyte ratio; PNI, prognostic nutritional index; ALC, absolute lymphocyte count; LMR, lymphocyte-to-monocyte ratio; A, albumin; AAR, albumin-to-alkaline phosphatase ratio; P, total protein; H, hemoglobin; SII-H, highest systemic inflammation index; SII-L, lowest systemic inflammation index; NLR-H, highest neutrophil-to-lymphocyte ratio; NLR-L, lowest neutrophil-to-lymphocyte ratio; PLR-H, highest platelet-to-lymphocyte ratio; PLR-L, lowest platelet-to-lymphocyte ratio; PNI-H, highest prognostic nutritional index; PNI-L, lowest prognostic nutritional index; ALC-L, lowest absolute lymphocyte count; LMR-H, highest lymphocyte-to-monocyte ratio; LMR-L, lowest lymphocyte-to-monocyte ratio; A-H, highest albumin; A-L, lowest albumin; AAR-H, highest albumin-to-alkaline phosphatase ratio; AAR-L, lowest albumin-to-alkaline phosphatase ratio; P-H, highest total protein; P-L, lowest total protein; H-H, highest hemoglobin; H-L, lowest hemoglobin.

**P* values were calculated using the Wilcoxon rank sum test
